# Reflections upon the Increase of Mental Unsoundness

**Published:** 1895-05

**Authors:** D. R. Wallace

**Affiliations:** Waco, Texas


### TEXAS MEDICAL JOURNAL.

ESTABLISHED JULY, 1885.

PUBLISHED MONTHLY.—SUBSCRIPTION $2.00 A YEAR.

VOL. X. AUSTIN, MAY, 1895. NO. 11.

### Original Contributions.

For Texas Medical Journal.

### REFLECTIONS UPON THE INCREASE OF MENTAL UNSOUNDNESS.

BY DR. D. R. WALLACE, M. D., LL. D., WACO, TEXAS.

Read before the Texas State Medical Association, at Dallas, April 24, 1895.

WERE it a matter of enough import to call for an explanation, I might state that this paper is indebted for its origin to the suggestion of friends, professional and unprofessional, with whom I have exchanged views upon the subject,—to lay before the profession certain opinions I have entertained now for years, upon the ætiology of mental unsoundness during the latter half, more particularly, of the nineteenth century. These views may be of little worth, but have not been hastily formed. They have grown up in my mind as the result of twenty years observation and of experience with the insane. Though much has been written upon the subject, little consensus of opinion has been reached; no more, perhaps, than existed forty-five years ago, when professional attention was called to it—I believe for the first time—by Dr. Kirkbride, of Philadelphia. It looks, to be sure, like carrying coal to Newcastle, or, to use a figure nearer home, like bringing artesian water to Waco, to be going over ground so thoroughly gleaned. For into what nook and corner of the subject have not Dick Hack Tuke, Mills, Mitchell, Hammond, Spitzka, and others, directed their researches? What

ætiological factor have they not tortured into telling all its secrets? What aspect of men or things have not Charcot, Comusat and Sapilli made the most of? Not to mention an army of other explorers on both sides of the ocean, as Lewis, Clouston, Brown, in England; Meynert, Crafft-Ebing and Mendel, of Germany; Voison, Luy, Achille and Forille, of France; Tamburani, Golgi and Mosilli, of Italy, and Kowalenski, of Russia.

Still there have been advanced for professional consideration and acceptance no explanation that explains the apparently contradictory phenomena connected with the increase of mental unsoundness during the period mentioned. It is not overstating or even straining the truth to say, the explorers among the brainiest, brawniest, most scientific workers of our times, have one and all retired from the quest, with the consciousness all too apparent that their labors were a failure, their efforts an abortion.

These reflections, it is needless to premise, are not intended to cover the whole field of the causation of mental unsoundness. Too vast a stretch of territory for one excursion,—the outlines, indeterminate as they are, certainly enclose too vast an area for one hasty survey. There is an outlying province of this bizarre, ill-defined domain that seems almost to have entirely escaped the researches and even observation of explorers. Upon this it is the purpose to fix the attention in these reflections,—modestly and rightly called theoretical, as not being susceptible, in the present state of our knowledge, of scientific verification.

There is a curious fact challenging attention at the threshold of the inquiry:—The period during which this increment in mental unsoundness has seemed most conspicuous, is just that in which the progress of the race in material development, in sciences, in the arts and appliances of civilized life, in the advancement of the institutions of civil government,—securing to the individual the most liberty and the greatest freedom compatible with public safety,—intellectual activity and moral power, have been most pronounced, in fact, unparalleled in the annals of the race.

There is another feature characterizing this period believed from the standpoint of these reflections significant. There has been an increasing unaccountable prevalence of crime in every stratum and walk of life.

Mental unsoundness in verbal strictness evinces its presence, it would seem, quite as often by outbreaks of crime as by breakdowns of madness. The oft quoted lines of Dryden as to the close relationship of "great wit and madness," would, it is believed, be nearer the truth, if made to read:

"Much crime to madness sure is close allied,
And thin partitions do their walls divide."

The criminal, a creature of his surroundings and associations, may not be discriminated from the man with mental disease. Indeed, it is not difficult to take the philanthropic position that all criminals are insane, because not in sympathy with the moral conceptions of their times—not in harmony with their environment. It goes without stating that it were not difficult to imagine conditions in which mental integrity, either intellectual or moral, would be impossible, as, on the other hand, it were easy to conceive of others in which mental unsoundness—madness or crime could hardly exist.

From statistics,—the most reliable within reach, and doubtless correct enough for all practical purposes,—there is, throughout Christendom, one insane to every four hundred of the population. In certain States of the American Union, notably in California, a mixed and motley population, and in Massachusetts, the most highly educated, the number of the insane to the population is much greater, being about one to every two hundred and fifty. Among the aborigines of America, insanity is seldom met with; in British India, among the native population it is rare; in Arabia, almost unknown. Insanity in China is estimated at one in every five thousand; including idiocy, one in every two thousand; while in Japan it is said that any form of mental impairment is seldom met with, except in those portions of country which have been longest and most subject to foreign influence. These figures will strike us as the more extraordinary when it is recollected how extensive the opium habit prevails, especially among the Chinese. All tolerably informed people know that the effects of the habit have been greatly exaggerated by Christian missionaries and others.

How is it we occidentals, Christians, appear to such disadvantage when compared with these heathen Chinese? Are not we Christians, especially we of the United States, the people? Have we not the greatest and best government on the planet—the only decent one, in fact? Our politicians tell us so, and they know. We have the only true religion, so our priests inform us. Their information is direct from the Deity, so there can be no mistake. We know it. True enough, no doubt, still the ugly fact glares out upon us from every part of our great country, that insanity is more common among us than elsewhere among men; and worse still, that it is alarmingly on the increase; while its congener, crime, follows hard after. The most thoughtful and best informed minds amongst us are almost, if not quite, ready to conclude that it is becoming so rampant as to seriously endanger the republican institutions of our glorious country and the greatest government in the world. It is certainly worth while to look into the condition of our country,—its religion, laws, manners and customs, habits of our people, the philosophy of life of our times. Peoples or nations, like individuals, pass through successive stages of development. They are born, grow up, grow old, decline, die. They have their youthful aspirations, manly convictions and ambitions, doubts and distrust, of seniscence and decrepitude. It may not be unprofitable to run a parallel between a republic, full of youthful aspirations and manly ambitions, and China, having run her career and now resting in her religion and philosophy, enjoying the calm and tranquility of age. Having passed through the phases of national life awaiting all peoples, a study of the manners and customs, institutions of government and religion of this remarkable people may serve to light up the intricacies and obscurities of our subject.

Marco Polo visited China, in company with his father, Nicolo, and uncle, Maffeo, in the 13th century, and published an account of his travels about six hundred years ago. The Polos were received with distinguished consideration and honor by Kublai Khan, the emperor, and Marco made governor of a great province. They were not only princes at home, but what was much better and more to the purpose, were educated in all the learning of their age and country. Hence they were very useful to Kublai Khan in regard to certain arts and phases of European civilization imperfectly or quite unknown in China, and were in turn given every opportunity to see the country and to become acquainted with the arts and sciences, religious institutions and philosophy of the country. They were finally permitted, but much against the wishes of Kublai Khan, to return to their own country loaded with presents of such value that we are assured there was not a royal exchequer in Europe that could have purchased the precious stones.

It is stated that on their return these enterprising Italians were received with distinction and accorded every honor except that of being believed when they related what they had seen. Even on his death bed, Marco was urged, in the name of religion, as he valued his soul, to retract his alleged falsehoods. But he reaffirmed them with his dying breath, and there is now no doubt that he spoke substantially the truth.

So far was China in advance of anything Europe had to show, Polo's account of Chinese civilization and material development was regarded by Europeans not only as incredible, but impossible, nor was it verified until the present century. Their immense gilded palaces and porcelain towers, their civil engineering in spanning vast rivers and ravines, tunneling through mountains, laying down aqueducts and canals with a skill hardly equalled in the west in our own times, were of course incredible to our savage ancestors of Europe. Even then the common people are described in China as dressing in brocade, silk and broadcloth, living in houses adorned with rich tapestry, when our ancestors were living in caves and hollow trees and mud hovels, and dressing in skins with wisps of straw about their legs.

With an area less than that of Europe, China contains a population estimated at over one-third of the human race. Their method, manners and customs, diet, social relations, institutions of government and religion, bottomed on an unchanging philosophy of life, have undergone no change for ages for the reason that long since these people attained to an ideal perfection in their civilization as adapted to their climatic condition and the genius of the race.

Dr. J. W. Draper, "Intellectual Development of Europe," an authority, says: "It demands the highest policy to govern populations living in great differences of latitude. Yet China, stretching over 20 degrees of latitude with a difference of temperature between the northern and southern provinces of 25 degrees F., has not only controlled her climatic strands of people, she has made them, if not homogeneous, yet so fitted to each other, that they all think and labor alike. The organization of the national intellect is the principle by which it is accomplished. A broad foundation is laid in universal education. The way to public advancement is open to all. Merit, as tested by repeated and the most searching competitive examinations is the passport to office." Our author adds: "The Chinese has heard of our discordant opinion, our intolerance toward those who differ in ideas from us, of our worship of wealth, of the honor we pay to birth; he has heard we sometimes commit political power to men so little above animals that they can neither read nor write; that we hold military success in esteem, and regard the profession of arms as the only suitable occupation of a gentleman. It is so long since his ancestors thought and acted in that manner, he justifies himself in regarding us as having hardly emerged from the barbarian stage."

To this picture limned by the cunning hand of the immortal Draper, I beg to add a patch or two of coloring from my own personal observation of these people, as I saw them some years ago on the Pacific slope. I was struck with their intelligence, efficiency and fidelity as laborers. Their dexterity is something wonderful. As servants I have seen nothing equal to them anywhere. I visited their banks, their vast mercantile houses, their Joss houses, their theatres, and even their banqueting halls. Such homogeneousness, such equality, had never entered my conceptions. The millionaire and laborer sat side by side dressed just alike,—the former putting on no airs of superiority; the latter showing no sign of conscious inferiority. The most perfect social equality exists between all classes. The Chinese coming to this country of course are from an inferior stratum of society, yet I think they are the most intelligent laborers we have. General Grant, in his trip around the world, mentions four very great men by whom he was particularly struck, Lord Beaconsfield, Prince Bismarck, Gambetta, and the Prime Minister of China, but he says, perhaps the greatest of them all was the prime minister of China. When it is remembered that the Chinese we see upon our western slope are from the least intelligent part of the population, one is struck with their fine cranial development, their quiet, immobile features and measured movements. Stolid want is less common among them, than among any other nationality; in fact, may be said to be unknown. They manifest a singular exemption from the ambitions, envies, jealousies and other corroding passions, unhappily so common among the Aryan races. What is more significant, as will be made more apparent in what follows, is the singular fact that they are perfectly satisfied with their philosophy of life and with their religion. The fundamental doctrines of their famous teachers, Confucius, Lao-tse, et al., it never occurs to them to question. Beset with no doubts, they leave the world with a quiet resignation unknown in other lands. The writer was told by the wife of a missionary, thirty years in China, "they seldom or never change their religion for that of the Christians;" that they would hang about the missions, seemed to be interested in religion, but it was only to obtain employment and be fed; and it was only the lowest class that would do this; that invariably when they came to die, as she had repeatedly witnessed, they would return to the faith of their fathers. "The only way," she added, "to make them Christians is to civilize them."

The United States has an area of over three millions of square miles,—stretches from north to south over 25 degrees of latitude, not including Alaska, and from east to west over 64 degrees of longitude, including every variety of soil, climate and scenery, inhabited by twenty-five or thirty civilized European and Asiatic nationalities, not to mention fifty or sixty aboriginal tribes with all their diversified feelings, notions, ideas and opinions, social, governmental and religious. Spanning this vast expanse from New York to San Francisco, from Chicago to New Orleans, from Portland, Maine, to San Diego, telegraphic communications make the whole one vast neighborhood, from every part of which at our breakfast tables, waiting for our steak and muffins, we read the doings and sayings. Out traveling the sun, we know three, five, seven thousand miles away the happenings at London, Paris, Rome, Vienna, Berlin, St. Petersburg, Pekin and Canton before, estimated by clock time, they actually take place. What an incongruous heterogeneous mass of discordant, intellectually indigestible material from all over the world is served up by the morning papers with our breakfast.

New York is nearer, to say nothing of the safety and comfort of travel, to San Francisco than it was to Boston a hundred years ago. Chicago travels to Cincinnati in eight hours, to New York in twenty, St. Louis in ten, to New Orleans, Galveston and San Antonio in forty-five, or to Seatle and San Francisco in seventy-two. As a result of such rapid intercommunication, what a motley commingling of peoples of all classes and nationalities! The race in the past have known nothing like it,—nothing approximating it. As little has mankind known in the past of the material development of our times, of the discoveries in the arts and sciences, comforts, conveniences and appliances of modern life. Old methods are gone;—new ones have come to take their places. There are new ways of doing old things;—new ones are being invented every day, originating new callings, trades and professions, with their new ideals and mechanical appliances,—thus changing or modifying all the economic relations of life. New facts, changed relations, new methods, heretofore untried fields of effort and striving, generating novel ideas, thoughts and sensation within the mind, stimulating the imagination and giving to it new wings on which to soar through the limitless regions of fancy, filling the soul with new hopes, ideals and aspirations, and with them, alas! every depraved appetite, passion and lust, incident to human nature. The appetite goads, passion corrodes, ambition rages, making their victim a pandemonium, the home of every unclean thing.

A word as to the development and functions of the brain,—"The dome of thought, the palace of the soul."

The practiced detective and others that find their account in such study and observation, are able to interpret the facial expression, the pose, conformation and movement of the body, so as to read the character, business, calling or profession of a given individual with almost unerring precision. Most persons can tell with a mere coup d'œil a man's nationality. As a rule, the Chinese all look alike in the main. Why? They have had the same or similar surroundings, the same scenery, been thinking the same thoughts, and doing the same things for thousands of years,—resulting in the establishment of, not only a corresponding facial expression, but of a structural development of the body as well. So true are the words of Tennyson,

> "I am a part of all I have met,"

and Byron's lines,

> "I live not in myself, but I become
> Portion of that around me"; or as Shakespeare puts it,

> "My nature is subdued
> To what it works in."

These facts are obvious to common observation; but though less obvious, there is another fact, far more potent over human destiny, viz.: these corporeal changes necessitating corresponding modification of the brain and whole nervous mechanism, result in commensurate changes in the character,—in the whole emotional, intellectual and moral man. Let it be borne in mind these changes come not in a day. They are secular—requiring ages for their accomplishment, and for them to come to a state of equilibrium. During the period of their coming, extending it may be through hundreds of generations, the brain, the most specialized tissue known to histology, and pro tanto unstable, is rendered by the process still more so, making it greatly more liable to take on diseased action.

Europe, during the millennium, from the 5th to the 15th century, desolated by religious and fanatical wars and bloodshed, followed by famine and pestilence, did not double its population. China, blessed with peace and quiet, and under the same imperial dynasty, doubled her population six times. We are accustomed to hear these harmless people called *heathens* and pagans. It may be that they are rightly so stigmatized. They are Buddhists. Having passed through the phases of their national life, as an individual passes from childhood to old age, they are now in a condition of national decrepitude and quietude. As a consequence they are not so progressive, certainly not so aggressive as Western peoples. It is believed, however, that they excel Aryan races in many very important particulars. To be sure, we are infinitely their superiors in many of the arts and sciences, especially those of war. We know how to kill each other a great deal better than they do, and we seem to take infinitely more pleasure in it than they do. In correspondence with their environment, they are physically, intellectually and morally at rest, as shown by the placid expression of their countenance, cranial development, dress, manners, customs, methods, institutions of government, art, religion and philosophy. They know nothing of the wild passions that distract the life, and maddening into fury, hurry on vast aggregations of people to war and bloodshed among occidental nations. In all these particulars more diversity than exists among the people of the United States, can hardly be imagined. The most supernormal occultism and the theosophy of India a thousand years ago, as interpreted and popularized in the writings of Madame Blavatsky, the positivism of Auguste Comte, and the bald materialism of Maudsley, Clifford and Frederick Harrison, in a word all forms of philosophy flourish side by side. Professing the simple faith of the humble Nazarine, every system of theology is equally welcome,—whether it be the Essenism of Origen, the Manichæism of Augustine, the dyspeptic pessimism of John Calvin, John Knox and Jonathan Edwards, the Armenianism of Wesley and Whitfield, the polygamous vagaries of Joe Smith and Brigham Young, or the wild rantings of the Salvation Army and the modern evangelist, it is of no consequence, our people are ready to run after it all the same.

In manners, customs and habits, ethics and government, opinion is equally unfixed, unstable. With no fixed system of philosophy, no accepted religion, our people are afloat at the mercy of the winds and waves without chart or compass, a starless vault overhead, on a shoreless sea, the great ocean of mortal life and human destiny,—the sport of chance and of every ignorant fanaticism. We have few beliefs, hardly any convictions, and no faith. What religion we have is wholly intellectual, is of the head and not of the heart. We persuade ourselves that we are Christians, but in the proper sense of religion we are not religious. We have religious institutions, not for the promotion of true religion, but for the upbuilding of the denominations. Our charities spring from the head, not the heart. The simple teachings of the Christ have been left behind in the way, or have been so stultified as to have lost their original spirit. They are well described by a Buddhist author, Mozoomdar, in a recent work entitled, The Oriental Christ. He says: "Was not Christ an Asiatic? He and his disciples were Asiatics, and all the agencies primarily employed for the propogation of the gospel were Asiatic. Yet the Christ that has been brought to us in India is an Englishman, with English manners and customs about him, and with a temper and spirit of the Englishman in him. Hence it is that the Hindu people shrink back. Go to the rising sun in the East, not to the setting sun in the West, if you wish to see Christ in the plentitude of his glory and in the fullness and freshness of the primitive dispensation. In England and Europe we find apostolical Christianity almost gone. We find the life of Christ formulated into lifeless forms and antiquidated systems. Look at this picture, and that:—this, the Christ of the East, that, of the West. When we speak of the Western Christ, we speak of the incarnation of theology, formalism and ethnical and physical force. When we speak of an Eastern Christ, we speak of the incarnation of unbounded love and grace." Such is the Christ from the oriental standpoint, we take as our great exemplar. We form intellectual conceptions of him and embody them in systems of theology, but true faith in him we have none. A few Sundays ago, a minister of the gospel standing in his pulpit in Boston said: "Do you know, my hearers, that all the diseases of the nervous system and brain are alarmingly on the increase?" He voiced the consensus of opinion throughout the so-called civilized world. Given the above conditions of the people of the United States, would not the enlightened psychologist, knowing the effect of physical agencies upon the physiological activities of man—upon his nervous system, his brain, his intellect, his affectional and emotional nature,—know, antecedent to all observation, that this must be the effect of the material development of our times? Nor would his prognosis stop here. He would foresee that such a people must be steeped in wickedness, guilty of every crime, actual or constructive, to which unrestrained selfishness can incite, or perverted emotion or blind passion, urge on its victims. That murder, suicide, robbery, dishonesty of every sort, have become so common as to hardly excite notice, we all know; but has it occurred to us that this is just what we should expect under the circumstances as the legitimate outcome of the conditions existing among us.

Philosophy is the guide of life. Religion purifies motive, and consoles grief. The people of this age and nation, stimulated to intense inordinate activity by the material developments of the time, are without the guidance of philosophy, or the purifying, supporting consolations of religion. In place of true philosophy we have the airy subtleties of metaphysics and the meaningless refinements of psychology. For the refining, ennobling, purifying, consoling influences of religion adapted to human nature, we have the hair-splittings of theology with about as much spiritual life in them as there is in a corpse. The masses of mankind are incapable of formulating a rational theory of the universe, that is to say, a theoretical system of the world. To them true religion is the more indispensable. May I ask, without offense to the religious sentiments of my countrymen, is not religion meeting the wants and aspirations and stage of development of the race, the great want of our times? The greatest preacher of our age and country, if not in the world, after having preached over fifty years, in one of the last sermons he ever delivered, makes this sad confession: "I am bound to say, that in the history of the world, while religious institutions have been valuable, and done a great deal of good, they have perhaps done as much harm as good. There is scarcely one single perversion of government, there is scarcely one persecution of men, there is scarcely a single one of the great wars that have depopulated the globe, there is scarcely one great heresy developed out of the tyranny of the church, that has not been the fruit of institutional religion; while that spirit of humanity that was to give the institution its motive power, has to a certain extent, died out of it."

To the philosophical inquirer, the cause is not far to seek nor hard to find. We have already alluded to the fact that a metaphysical theology has taken the place of a religion of simple faith. "The Autocrat of the Breakfast Table," though in a humorous way, tells us many stern truths, none more so, than when he says: "Insanity is often the logic of an accurate mind overtasked. Good mental machinery ought to break its own wheels and levers if anything is thrust among them suddenly, which tends to stop them or reverse their motion. We frequently see persons in insane hospitals, sent there in consequence of what are called religious mental disturbances. I confess that I think better of them than of many who hold the same notions and keep their wits and appear to enjoy life very well outside of the asylums. Any decent person ought to go mad if he really holds such or such opinions. It is very much to his discredit in every point of view if he does not. Perhaps more than one of you hold such as I should think ought to send you straight over to Summerville, if you have any logic in your head, or any human feeling in your hearts. Anything that is brutal, cruel, heathenish, that makes life hopeless for the most of mankind, and for entire races, anything that assumes the necessity of the extermination of instinct, which was given to be regulated—no matter by what name you call it—no matter whether a fakir, or a monk, or deacon believes it—if received, ought to produce insanity in every regulated mind. That condition becomes a normal one under the circumstances. I am very much ashamed of some people for retaining their reason when they know perfectly well that if they were not the most stupid or the most selfish of human beings, they would go mad at once."

In this playful paragraph the great man who has recently left us, full of years and honors, suggests more of the real cause of the increase of mental unsoundness of our times than is to be found in ponderous tomes that might be named, written to explain it.

To those who most need the consolations and support of religion in the struggles of life—in times of darkness and gloom—in their bereavement when earth-born hope is dead and they know not where to look, the ministrations of institutional religion or theology, are dead-sea apples, turning to ashes in the grasp of grief and sorrow. By the cultured and learned, capable of hair-splitting metaphysico-verbal distinctions, these theological teachings—such of them as are plainly at war with the common sense and experience of mankind—are refined away. By the extremely low, depraved and ignorant they are unheeded. It is upon the middle class, with a corresponding amount of intelligence and virtue, who want to know the right and to do their duty, upon whom these man-made systems of theology, emptied of all religion, fall with most deplorable, disastrous effects. The amount of misery thus inflicted upon the most numerous and worthy portion of the human race, we may never know; but we may form some idea, may make a guess approximating the reality, when we inspect the statistics of lunatic hospitals and see what numbers of these plain, good people—with whom words have no meaning other than that attached to them in the every-day intercourse of life; with whom a spade is a spade, are within the walls of these institution. Think of it, this is just the class one would suppose would suffer least from mental unsoundness of any kind, being free from pinching want, carking cares, corroding passions, exciting, maddening ambitions—causes, popularly thought to be most productive of this class of troubles. The stratum of society below furnishes the criminals, as a rule. This is the general tendency—exceptions, of course. The lower stratum contributes to the criminal class, the middle to the insane class, the upper to both. Once and again an intellectual giant, like Hugh Miller, of "Old Red Sand Stone" memory, is forced by his theology so far out of his rapport with common sense and experience as to fall a victim to insanity and suicide; while occasionally a good and unlettered man is frightened by the hobgoblins of mediæval dreamers from the moorings of reason into the darkness of insanity.

Though not carried out on all the lines intended, as the survey is assuming proportions not contemplated, I close. If the above is true, or even an approximation to the truth, it is not difficult to see why mental unsoundness is on the increase, at least, in this age and country.

For the imperfections of execution in the paper, I bespeak your indulgence; for its shortcomings generally, your kindly criticism, tempered with charity.

"The more I think of it," says Mr. Ruskin, "I find this conclusion the more impressed upon me:—that the greatest thing a human soul ever does in this world is to see something, and to tell what he saw, in a plain way." I have tried to see something along the lines indicated and have thought I saw it. I thought I saw,—it may have been illusion—a mirage. I need not remind you how difficult it is for one to get himself down on paper. We need not the genius of a Lord Bulwer Lytton, to tell us: "Writing, after all, is a cold, coarse interpreter of thought. How much of the imagination, how much of the intellect is lost while we seek to embody it in words." Our own Holmes, the Poet Laureate of the medical profession of the United States, has versified the idea in the lines—

"Our whitest pearls we never find,
Our ripest fruit we never reach,
The flowering moments of the mind
Drop half their petals in our speech."

